# Correspondence Between Resting-State and Episodic Memory-Task Related Networks in Elderly Subjects

**DOI:** 10.3389/fnagi.2018.00362

**Published:** 2018-11-08

**Authors:** Lee Simon-Vermot, Alexander N. W. Taylor, Miguel À. Araque Caballero, Nicolai Franzmeier, Katharina Buerger, Cihan Catak, Daniel Janowitz, Lana M. Kambeitz-Ilankovic, Birgit Ertl-Wagner, Marco Duering, Michael Ewers

**Affiliations:** ^1^Institute for Stroke and Dementia Research, Klinikum der Universität München, Ludwig Maximilian University, Munich, Germany; ^2^Department of Psychology, Aberystwyth University, Aberystwyth, United Kingdom; ^3^German Center for Neurodegenerative Diseases, Munich, Germany; ^4^Department of Psychiatry and Psychotherapy, Ludwig Maximilian University, Munich, Germany; ^5^Institute for Clinical Radiology, Klinikum der Universität München, Ludwig Maximilian University, Munich, Germany

**Keywords:** resting-state fMRI, brain activation, episodic memory, connectivity, network

## Abstract

Resting-state fMRI studies demonstrated temporally synchronous fluctuations in brain activity among ensembles of brain regions, suggesting the existence of intrinsic functional networks. A spatial match between some of the resting-state networks and regional brain activation during cognitive tasks has been noted, suggesting that resting-state networks support particular cognitive abilities. However, the spatial match and predictive value of any resting-state network and regional brain activation during episodic memory is only poorly understood. In order to address this research gap, we obtained fMRI acquired both during rest and a face-name association task in 38 healthy elderly subjects. In separate independent component analyses, networks of correlated brain activity during rest or the episodic memory task were identified. For the independent components identified for task-based fMRI, the design matrix of successful encoding or retrieval trials was regressed against the time course of each of the component to identify significantly activated networks. Spatial regression was used to assess the match of resting-state networks against those related to successful memory encoding or retrieval. We found that resting-state networks covering the medial temporal, middle temporal, and frontal areas showed increased activity during successful encoding. Resting-state networks located within posterior brain regions showed increased activity during successful recognition. However, the level of resting-state network connectivity was not predictive of the task-related activity in these networks. These results suggest that a circumscribed number of functional networks detectable during rest become engaged during successful episodic memory. However, higher intrinsic connectivity at rest may not translate into higher network expression during episodic memory.

## Introduction

Functional connectivity (FC) designates the correlation of brain activity between different brain regions. Functional MRI (rsfMRI) of BOLD signal changes obtained during resting-state, i.e., when a subject is not engaged by a particular cognitive stimulation, demonstrated FC between different brain regions. Regions that show high FC between each other are thought to form functional networks, where rsfMRI studies have identified several large-scale resting-state networks in the brain (Damoiseaux et al., 2006). Since rsfMRI is obtained without overt cognitive performance, the role of resting-state networks in cognitive processes is still an open question (Shirer et al., 2012; Elton and Gao, 2015). Meta-analysis of a large number fMRI studies showed task-related co-activation patterns mapped onto major resting-state networks (Smith et al., 2009; Di et al., 2013), suggesting that regions intrinsically connected during resting-state become simultaneously activated during tasks. Several studies assessing FC during both resting-state and task-related fMRI in young healthy subjects have largely confirmed such a hypothesis for a variety of cognitive tasks (Greicius et al., 2003; Calhoun et al., 2008; Andrews-Hanna et al., 2010; Cole et al., 2014). In fact, rsfMRI activity levels in the brain were found together with morphological brain differences to be predictive of the spatial pattern of brain activation during perception and higher cognitive abilities such as language and working memory (Tavor et al., 2016). These studies suggest a spatial match between resting-state networks and those patterns of task related brain activation. Most previous combined resting-state and task-related fMRI studies focused on tasks based on visual or auditory perception (Arfanakis et al., 2000; Bartels and Zeki, 2005; Fair et al., 2007; Cole et al., 2014; Elton and Gao, 2015; Tavor et al., 2016), motor function (Arfanakis et al., 2000; Jiang et al., 2004; Morgan and Price, 2004; Cole et al., 2014; Ganger et al., 2015; Tavor et al., 2016), attention (Calhoun et al., 2008; Hellyer et al., 2014; Tomasi et al., 2014), language (Arfanakis et al., 2000; Fair et al., 2007; Hampson et al., 2010; Cole et al., 2014; Elton and Gao, 2015), or working memory function (Fransson, 2006; Cole et al., 2014; Elton and Gao, 2015; Tavor et al., 2016). Strikingly there is a dearth of studies testing the match between episodic memory related networks and resting-state networks. Huijbers et al. (2013) assessed in which resting-state networks activation peaks obtained during an episodic memory task fall, but did not attempt to test which resting-state networks showed task-related connectivity. A possible explanation for the lack of studies is the fact that none of the canonical set of large-scale resting-state networks corresponds to known patterns of episodic memory processes (Smith et al., 2009). From a clinical point of view, the establishment of a match between resting-state and episodic memory related network connectivity is of great importance to assess network failure underlying memory impairment in aging and neurodegenerative disease including Alzheimer’s disease (Meskaldji et al., 2016; Zhang et al., 2016). In order to address this research gap, we assessed fMRI during both rsfMRI and an episodic memory task including face-name association learning in cognitively healthy elderly subjects. Specifically, using independent component analysis (ICA) (Calhoun et al., 2001), we assessed the association between functional networks related to successful encoding or recognition and resting-state networks. In addition to testing the spatial match between task-related networks and resting-state networks, we assessed whether resting-state component values are predictive of the level of the task-related network expression during successful encoding or successful recognition. We hypothesized that especially medial temporal components show a match between resting-state and memory task-related networks. Secondly, we hypothesized that the level of resting-state networks is predictive of the level of task-related network connectivity in medial temporal lobe components.

## Materials and Methods

### Participants

A total of 38 elderly cognitively healthy participants (HC) were included. All subjects were recruited at the Memory Clinic of the Institute for Stroke and Dementia Research (Klinikum der Universität München, Germany). Inclusion criteria were: Age > 60 years, cognitive performance within 1.5 SD of age- and education-adjusted norms of all neuropsychological tests included in the Consortium to Establish a Registry for Alzheimer’s Disease (CERAD)-Plus battery (Schmid et al., 2014). Exclusion criteria were: Presence of depressive symptoms, evidence of other acute or past neurological/psychiatric disorders, history of drug or alcohol abuse, diabetes mellitus, premorbid IQ < 85, and MRI contraindications such as presence of ferromagnetic implants, pacemakers, or cochlear implants. The cognitively normal subjects were either relatives of patients, were recruited during information events at nursing homes and open day events of the institute, or came in response to news articles or because of subjective cognitive complaints. Subjective cognitive decline (SCD) was defined by a participant’s complaints about worsening of cognitive abilities such as memory that started to occur at any time during the last 5 years. The presence of SCD and the neuropsychological profile is presented in Table 1. The participants’ assessment was completed in two visits: on the first day, the subjects underwent a neuropsychological and physical examination, followed by a structural MRI (T1 MPRAGE, FLAIR, DTI) and a rsfMRI. On the second day, the participants performed a face-name association task fMRI. In some subjects (*n* = 8), the rsfMRI was acquired 1–12 weeks after the task-fMRI due to inconvenience of prolonged data acquisition on day 1. The study was approved by the ethics committee of the Ludwig Maximilian University, Munich. All participants provided written informed consent.

**Table 1:** Participants’ characteristics indicated as the mean and standard deviation (in bracket) for continuous variables.

	Cognitively normal elderly subjects (*n* = 38)
Age	72.5 (5.78)
Years of education	13.61 (3.04)
Subject cognitive decline (yes/no)	11/27
MMSE	29.34 (0.91)
CERAD word list – delayed free recall	8.21 (1.36)
Verbal fluency animals	24.5 (5.13)
Boston naming test	14.47 (0.72)

### MRI Parameters

All MRI scans were obtained on a Siemens Verio 3T MRI scanner. The functional task was acquired with a 12 channel head coil and a T2^∗^-weighted echo-planar imaging (EPI) pulse sequence with 3 mm × 3.4 mm × 3.4 mm slices [inter-slice gap = 1 mm; echo time (TE) = 30 ms, repetition time (TR) = 2000 ms; flip angle = 90°; parallel acquisition (GRAPPA) with acceleration factor 2; field of view (FOV) = 220 mm × 220 mm; 64 × 64 data acquisition matrix]. A high-resolution MPRAGE T1-weighted sequence with 1 mm slices in the sagittal plain [interval time (TI) = 900 ms; TE = 2.52 ms; TR = 1750 ms; Flip angel = 90°; phasing encoding anterior to posterior; FOV = 256 mm × 256 mm; matrix = 246 × 256; single acquisition] was used for the structural image. Field maps were acquired to enable the *post hoc* correction of susceptibility artifacts (same parameters as the EPI, TE = 4.92/7.38 ms, TR = 488 ms, and flip angle = 60°). For the resting-state fMRI, a 32 channel head coil (day 1 visit) was used, with the acquisition parameters consisting of a T2^∗^-weighted EPI pulse sequence with 3.5 mm voxel resolution. The overall scan comprised 180 volumes prior to which subjects were instructed to keep their eyes closed.

### fMRI Memory Task

The face-name task contained 112 encoding and 112 recognition trials, divided into 14 blocks of face-name encoding (of 8 trials), each followed by a recognition block (of 8 trials). A total of 112 different faces were used (½ female, ½ male) from the Glasgow Unfamiliar Face Database^1^. The criteria of selection for faces were direct gaze, European ethnicity, neutral expression and no face jewelry or hair accessories to standardize the facial features across different images. One sixty eight different names (½ female, ½ male) were selected from the Leipzig Corpora Collection^2^ matched for character length (5 or 6 letters) and frequency of occurrences. During an encoding trial, a photo of a face and a first name shown below were presented and the participant was instructed to learn the name belonging to the particular person shown. During the subsequent recognition block, the faces previously seen in the encoding block were presented again, but this time together with two juxtaposed names, one correct and one distractor. The participant had to decide, via left or right button press, which of the two names had been presented previously with that face. In each recognition trial, the presented distractor could be either a new name (that had never been seen before, *n* = 56 trials) or a name (that had been associated with another face in the previous encoding block, *n* = 56 trials). Each stimulus was presented for 5 s with a randomized inter-trial-interval of 1500–3000 ms between trials through vision goggles attached to the head coil, which could be corrected for individual eyesight differences. The whole task took about 30 min to complete. The classification of encoding trials as successful or unsuccessful was determined based on whether the corresponding face-name pair was correctly recalled. The ratio of successfully recalled trials relative to the total amount of trials was computed to quantify a subject’s task performance. All participants were familiarized with the face-name task by a brief test trial on a laptop conducted outside the scanner before the fMRI session commenced.

### fMRI Preprocessing

The preprocessing was done using SPM12 (Wellcome Trust Centre for Neuroimaging, UCL, London, United Kingdom). All images [T1 and EPI (task and rest) and field map images] were manually reoriented to the anterior commissure and angled to the posterior commissure. The T1-weighted MPRAGE scans were segmented into gray matter (GM), white matter (WM), and cerebro-spinal fluid (CSF) maps. The diffeomorphic high dimensional transformations were estimated based on the three segments using the DARTEL tool implemented in SPM12. The resulting GM group template was coregistered to the (affine) MNI template in SPM12 and the two transformation matrices (high-dimensional and affine) were combined for spatial normalization into the MNI space.

The task and resting-state EPI images were slice-time corrected, realigned, and unwrapped applying the field map to account for scanner inhomogeneity variations. None of the subjects’ motion parameters were larger than 3 mm translation or two degrees rotation. Subsequently, for each participant, the images were coregistered to the individual’s T1 image and normalized to MNI space by applying the transformation parameters estimated through DARTEL. An 8 mm Full width half maximum (FWHM) smoothing kernel was applied and the smoothed images were resampled to 1.5 mm voxel resolution. For resting-state images only, a linear trend was removed and a band pass filter was applied to remove frequencies between 0.01 and 0.08 Hz. WM and CSF signal were regressed out of the time series voxel by voxel.

### Task-Related fMRI Activation

A fixed-effects general linear model was used to test increased activation during correct vs. incorrect trials of encoding or recognition. We created the regressors with time onsets for each stimulus presentation and convolved the time series with a canonical hemodynamic function, including six motions parameters, temporal, and dispersion derivatives. Six regressors were included in the model (successful encoding, unsuccessful encoding, successful recognition, unsuccessful recognition, encoding instructions, and recognition instructions). The regression models were computed at subject level for subsequent group analyses (see Statistics below). The univariate group level analysis of activation during successful encoding and retrieval have been reported elsewhere (Franzmeier et al., 2017).

### fMRI Based Network Analysis via ICA

We applied group ICA to decompose the fMRI data into a set of components, where spatial independence between components is defined based on maximizing the independence of the voxel-based BOLD time series between sets of voxels. The GIFT toolbox (GroupICAT v4.0a^3^) was used to perform such a group spatial ICA using the Infomax algorithm (Bell and Sejnowski, 1995), separately for task fMRI and rsfMRI. The image time courses were scaled to the same global mean by extracting the mean per time point from each volume as implemented in GIFT. For the task fMRI, we used the minimum description length algorithm (MDL) to estimate the ideal number of spatially independent components (IC, *n* = 24) (Li et al., 2007). The ICA was repeated 20 times using ICASSO (Himberg et al., 2004), to verify that the component estimates were stable. The subject-specific spatial maps and associated time courses were generated via back-reconstruction using the GICA3 method (Himberg et al., 2004). For rsfMRI, we applied group ICA, using the same parameters and number of ICs (*n* = 24).

### Statistical Analysis

In order to assess the spatial match between ICs that were significantly related to successful encoding and recognition of face-name pairs and any of the resting-state ICs, we conducted the statistical analysis in several steps. For ICs derived from the task-related fMRI scans, regression analyses were conducted to assess the association between a component’s time course and the task-design matrix, employing the GIFT toolbox. To this end, regressors including the temporal onset and duration of each stimulus were constructed for each of the four trial types: “successful encoding,” “unsuccessful encoding,” “successful recognition,” and “unsuccessful recognition.” This resulted for each participant into 4 trial-type specific beta-coefficients for each component. Secondly, in order to test which component’s time course was significantly associated with correct encoding or correct recognition, we applied a one-sample *t*-test to the corresponding beta-weights across subjects. Before computing the two-sample *t*-tests, all data were checked for outliers and removed if the standard deviation was larger than 3. The normal distribution of the data was tested with the Shapiro test. The Shapiro test was significant for successful recognition for the cerebellar-occipital network, suggesting non-normal distribution. For that network, a Wilcoxon signed rank test was used. For those components that showed a significant association of the BOLD signal with stimulus presentation during either correct encoding or correct recognition, we subsequently tested via two-sample *t*-test if the association was higher for successful vs. unsuccessful condition. The statistical analyses were conducted with the “stats” package (version 3.2.2) included in R^4^ (R-Core-Team, 2017). Lastly, in order to spatially match the thus determined task-related components of either successful encoding or recognition against the rsfMRI components, we conducted spatial regression analyses between each pair of any of those task-related and each of the resting-state IC maps. That is, Pearson product-moment correlations between the *z*-score transformed IC maps were conducted. Based on spatial correlation between a task-related fMRI component and any rsfMRI component, the spatial match was determined. The best match is reported. A unique match was possible in most cases due to a dramatic drop in the Pearson-moment correlation by >36% for the second-best matching component. See results for specifics. Next, for validation purposes, we tested whether any of the identified components corresponded to previously established resting-state network templates including 10 canonical ICs from a low-dimensional ICA and 70 ICs from a high-dimensional ICA (Smith et al., 2009). The spatial overlap was quantified (1) by spatial regression, and (2) Dice similarity coefficient based on our binarized ICs (thresholded at *z* > 2) and the external resting-state IC templates (thresholded at *z* > 3). For each pair of ICs, the Dice coefficient was computed as the ratio of the number of voxels within overlapping regions for a given pair of ICs and the total number of voxels. The dice coefficient can be interpreted as following: <0.2 poor, 0.2–0.4 fair, 0.4–0.6 moderate, 0.6–0.8 good, and >0.8 excellent correspondence. The spatial correlations and associated Dice coefficients were computed using our own MATLAB scripts. Bonferroni correction was applied on the spatial correlations to correct for multiple testing.

Next, we aimed to examine the value of the expression of a resting-state IC for predicting the degree of task-related activity of the spatially corresponding IC. To this end, we conducted a linear regression analyses according to: Y_i_ ≈ X_i_ + Age_i_ + Gender_i_ + ε. Where Y_i_ is a subject’s beta-value of a given task-related IC and X_i_ is a subject’s beta-value of the spatially matching resting-state IC. Thus, we tested to what extent the activation of an IC during successful encoding or recall (Y_i_, i.e., the correlation of the i^th^ subject’s IC time course with the trial-type design matrix) is associated with the degree of FC of the spatially matching resting-state component (X_i,_ i.e., the correlation between the i^th^ subject’s IC time course with that of the average group-level time course of that IC). All the linear models were computed using the “lm” command implemented in *R* (R-Core-Team, 2017). We corrected for type-1 error due to multiple comparison by applying Bonferroni correction.

## Results

Demographics details are displayed in Table 1.

### ICA-Based Network Activity During Successful Encoding and Recognition

**FIGURE 1:**
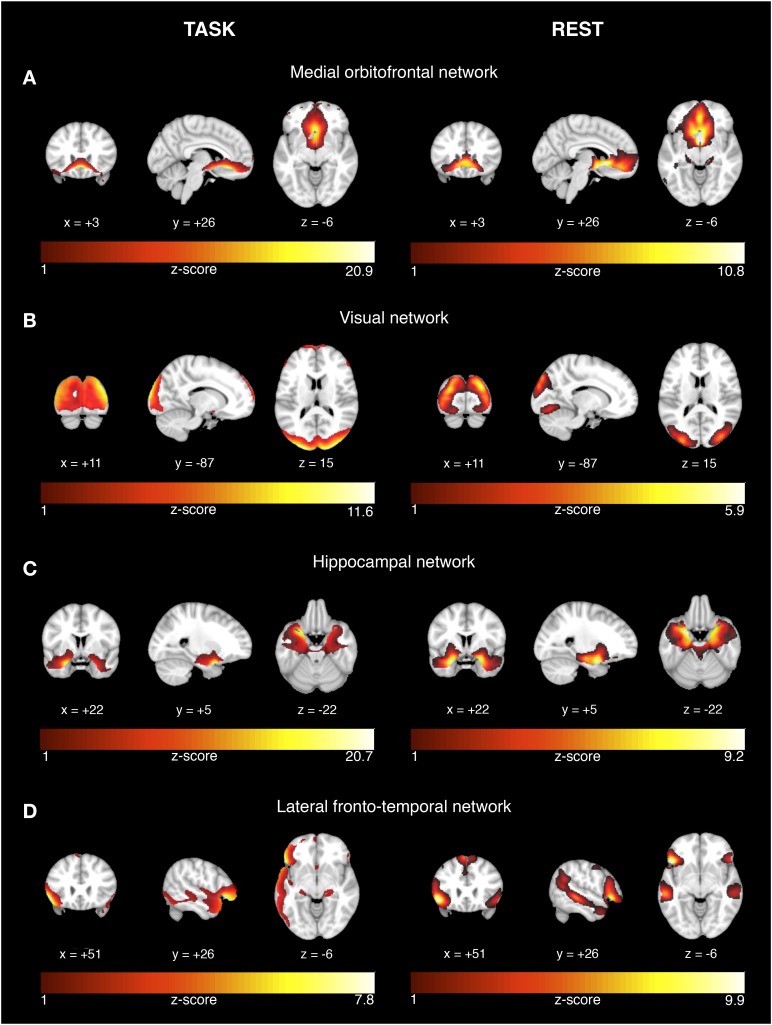
Network activation during successful encoding memory task and corresponding networks during rest. The results are displayed on representative sections of the 152 MNI template (1 mm resolution) for each functional network (rows **A–D**) derived from task-related fMRI (left panel) and resting-state fMRI (right panel). The task-related networks are thresholded at *z* > 2 and the rsfMRI were thresholded at *z* > 3. The color bars indicate *z*-scores. On the coronal and axial views, the left side of the image corresponds to the left brain side.

**FIGURE 2:**
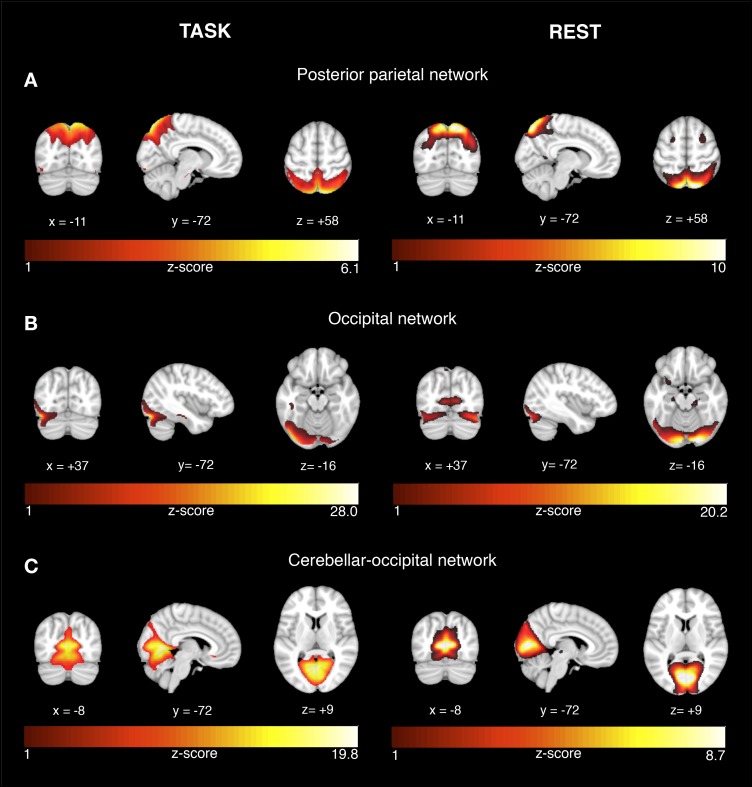
Network activation during successful recognition memory task and corresponding networks during rest. The results are displayed on representative sections of the 152 MNI template (1 mm resolution) for each functional network (rows **A–C**) derived from task-related fMRI (left panel) and resting-state fMRI (right panel). The task-related networks are thresholded at *z* > 2 and the rsfMRI were thresholded at *z* > 3. The color bars indicate z-scores. On the coronal and axial views, the left side of the image corresponds to the left brain side.

Among the 24 estimated ICs, we identified those ICs that showed significantly higher expression during successful vs. unsuccessful encoding or recognition trials. For encoding, four ICs showed higher task-related activity during successful vs. unsuccessful trials (a) medial orbitofrontal network [*t*(37) = 2.0, *p* = 0.026], (b) visual network [t(37) = 7.91, *p* < 0.0001], (c) hippocampal network [*t*(37) = 3.85, *p* < 0.001], and (d) lateral temporal-frontal network [*t*(37)2.74, *p* = 0.0047] (Figure 1, left panel). For recognition, three ICs showed higher task-related BOLD signal variation during successful vs. unsuccessful trials for: (a) posterior parietal network [*t*(37) = 1.84, *p* = 0.037], (b) occipital network [*t*(36) = 1.98, *p* = 0.025], and (c) cerebellar-occipital network [Wilcoxon signed rank test: V(36) = 561, *p* = 0.0006, Figure 2, left panel]. In order to allow for sufficient statistical power, these tests were not corrected for multiple comparisons.

### Spatial Correspondence Between Task-Related and Resting-State Networks

For each the four task-related components associated with successful encoding, spatial regression analysis showed a unique match to a particular rsfMRI component, including a medial orbitofrontal component (*r* = 0.68, *p* < 0.0001, Figure 1A), visual component (*r* = 0.61, *p* < 0.0001, Figure 1B), the hippocampal component (*r* = 0.74, *p* < 0.0001, Figure 1C), and the lateral fronto-temporal component (*r* = 0.39, *p* < 0.0001, Figure 1D). For any of these task-related components, the correlation coefficient of the second-best matching rsfMRI was >36%, providing a clear unambiguous match between the best matching components. For the task-related components associated with successful recognition, spatial regression analysis showed a unique match to rsfMRI components for the posterior parietal network (*r* = 0.64, *p* < 0.00001, Figure 2A), the occipital network (*r* = 0.45, *p* < 0.0001, Figure 2B), and the cerebellar-occipital network (*r* = 0.57, *p* < 0.0001, Figure 2C). Excluding SCD subjects from the spatial correlation analysis yielded virtually the same results (data not shown), suggesting that presence of SCD did not influence the findings. A unique match between a task-related component and the rsfMRI components was present for each of these components (drop in correlation value for the second-best matching rsfMRI component was >40%), except for the cerebellar-occipital network. For the latter network, second-best matching components covered also cerebellar-occipital areas, but reached into more anterior occipital/and subcortical regions not covered by the task-related component (correlation coefficient of *r* = 0.41, Supplementary Figure 1). All presented *p*-values are Bonferroni corrected for multiple testing.

**FIGURE 3:**
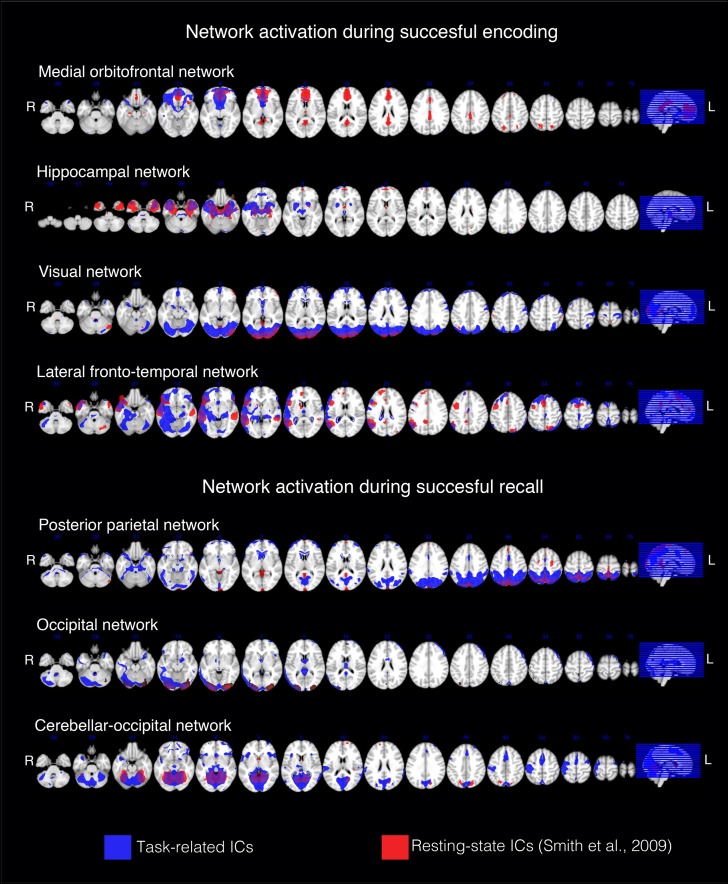
Spatial correspondence between each of the successful-encoding/recognition related ICs from the current study and the resting-state ICs from the 70 component ICA previously reported (Smith et al., 2009). The results are displayed on representative sections of the 152 MNI template (1 mm resolution). The task-related networks are thresholded at *z* > 2 and binarized. The Smith rsfMRI IC maps were thresholded at *z* > 3 and binarized.

To test whether the spatial correlation found between task-associated networks and resting-state network generalizes to resting-state networks found in an independent cohort, we computed the spatial correlation between our task-related ICs (the four matched successful encoding and three matched successful recognition related ICs) and each of the ICA-derived resting-state networks reported previously by another group (Smith et al., 2009). Smith et al. (2009) reported two template sets including either *n* = 10 ICs or *n* = 70 ICs. For the set of large-scale networks (*n* = 10 ICs), spatial regression yielded no significant spatial similarity with any of the successful-encoding/recognition related ICs (*p* > 0.05). However, for the 70 component resting-state ICA, each of the seven successful-encoding/recognition related ICs matched a resting-state IC (*p* < 0.0001, Figure 3 and Table 2).

**Table 2:** Dice coefficient and spatial correlation between functional networks activated during successful episodic memory (encoding or recognition) and the best matching networks from 70 resting-state maps (Smith et al., 2009).

Task networks	Dice coefficient	Dice coefficient. rating	Spatial correlation coefficient	*p*-Value
**Successful Encoding**				
Medial orbitofrontal	0.29588	Fair	0.299	<0.001
Visual	0.68855	Moderate	0.472	<0.001
Hippocampal	1.0913	Excellent	0.538	<0.001
Lateral fronto-temporal	0.39746	Fair	0.365	<0.001
**Successful Recognition**				
Posterior parietal	0.52081	Moderate	0.398	<0.001
Occipital	0.44051	Moderate	0.348	<0.001
Cerebellar-occipital network	0.82164	Excellent	0.541	<0.001

### Prediction of Network Activity During Successful Encoding and Recognition Based on rsfMRI Network Expression

We tested whether a subject’s network activity during successful performance on the episodic memory task could be predicted a subject’s the level of expression of the spatially corresponding resting-state network. No associations were found (*p* > 0.05).

## Discussion

The major findings of the current study were that task-related activity of (1) networks within the medial and lateral temporal lobe, occipital lobe and medial frontal cortex were associated with successful memory encoding and (2) networks primarily within the posterior parietal and occipital brain regions were associated with successful memory recognition. Each of these networks showed a spatial match to resting-state networks. However, higher resting-state connectivity did not predict higher task-related network activity for these networks. Together these results suggest that particular resting-state networks become engaged during successful episodic memory, although the strength of resting-state connectivity of a functional network is not predictive of the level expression of that network during episodic memory.

Our first findings showed that particularly hippocampal, lateral temporal, and frontal networks were engaged during successful encoding but posterior parietal networks were engaged during successful recognition. These results are largely consistent with those of a recent meta-analysis of brain activation during episodic-memory task, demonstrating increased activation of the hippocampus, lateral prefrontal cortex, and lateral temporal brain areas during encoding, hippocampal, and posterior parietal activation during recognition memory (Kim, 2015). Our findings are also consistent with previous findings of successful encoding-related hippocampus activity during face-name association learning in young subjects (Sperling et al., 2003; Zeineh et al., 2003) and cognitively healthy older subjects (Pariente et al., 2005). Our findings of posterior parietal networks being specifically engaged during successful recognition but not encoding is consistent with the previous proposed encoding/retrieval flip hypothesis of stronger engagement of the posterior parietal brain regions during retrieval compared to encoding (Daselaar et al., 2009). In contrast, we found occipital networks being associated with both successful encoding and recognition owing to the visual presentation of the stimuli on both conditions of the face-name association task. Together, the current findings of the ICA based analysis of task-related brain activity recapitulates largely previous fMRI activation studies on episodic memory.

For our second finding, we identified for each task-related network a unique match of a resting-state network. Importantly, in the current study those networks obtained during both task and rest corresponded to resting-state network components previously reported in an independent study using high dimensional ICA (i.e., *n* = 70 estimated ICs). In contrast, no significant overlap was found with large-scale networks, although a partial overlap with the DMN was evident for the medial temporal network during encoding and the cerebellar-occipital network during recognition. These findings suggest that smaller functional clusters rather than the entire large-scale networks are recruited during successful episodic memory. Our findings of such a spatially circumscribed successful memory related FC also explains why the matching of large-scale resting-state networks to episodic memory related patterns of brain activity among the canonical set of resting-state networks has been difficult so far (Smith et al., 2009). Large-scale networks such as the DMN and fronto-parietal controls networks are not singular networks but heterogeneous in nature (Cole and Schneider, 2007; Power et al., 2011), containing several distinct subcomponents where each one supports different cognitive functions (Cole and Schneider, 2007; Cole et al., 2013). Subcomponents may be selectively activated during memory (Shirer et al., 2012) and couple across different large-scale networks in a task-dependent manner (Bassett et al., 2011). For the DMN regions, we found that task-related network activity during successful retrieval overlapped with DMN only in posterior parietal regions. This is consistent with previous findings of the posterior parietal brain regions to be selective for successful retrieval of more “objective” facts (for meta-analysis see Spaniol et al., 2009), such as those tapped by the current recognition task of face-name pairs. In contrast, previous findings on autobiographical memory, i.e., memory of more personal events, have been found in both anterior and posterior regions of the DMN (Spreng and Grady, 2010; Elton and Gao, 2015). The selective involvement of the anterior medial frontal DMN may be specifically required for supporting self-referential processes during autobiographic memory (Andrews-Hanna et al., 2010; Sestieri et al., 2011). Together the current findings suggest the involvement of intrinsically wired networks that depart from large-scale canonical networks and match smaller clusters that are selectively recruited during successful episodic memory encoding and retrieval.

For our third result, we did not find the level of connectivity during resting-state to be predictive of the level of task-related connectivity. Note that this approach is fundamentally different from identifying intrinsic networks that may be recruited during a task (i.e., finding a spatial match). Instead, the strength of resting-state connectivity is probed as a predictor of task-related network “activation.” Results from a seminal previous study suggested that the task-induced activation is the additive combination of ongoing resting-state network connectivity and task-specific recruitment of neural activity (Fox et al., 2006). In fact, during a finger-tapping task that led to unilateral motor cortex activation, FC of the non-activated contra-lateral motor cortex explained over 85% of the task-related activity in the activated side of the motor cortex (Fox et al., 2006). The current results are not in conflict with these previous results; rather they suggest that a higher resting-state connectivity *per se* does not translate into higher task-related synchronization of brain activity in that network. A recent study reported resting-state network connectivity to be predictive of task-related activity (Tavor et al., 2016). However, it is important to note that only the spatial extent and distribution of task-related brain activity was assessed but not the level of task-related connectivity or activation. Thus, the predictive power reported in that previous study derives mostly from the spatial match between resting-state and task-related networks.

For the interpretation of the current results, some caveats must be taken into consideration. First of all, the sample size was relatively small and the results need to be replicated in a larger sample of studies. Still, we showed that the spatial match between encoding/recognition related ICs and resting-state IC could be generalized to resting-state ICs derived from an independent study, which suggests that the ICs were unlikely to be confounded by group specific characteristics. Secondly, we assessed task-related network activity by first computing the ICA and subsequently determining the association between an ICs time course to the task design. The component values can be considered a measure of FC of such task-related ICs. However, alternative measures that assess the change in FC due to task-stimulation is psycho-physiological interaction (PPI) analysis. This may be more sensitive to assess task-related FC. However, the PPI approach has been primarily tailored for the assessment of FC changes of single (seed) regions. It is thus difficult to apply to the whole brain for the identification of large-scale network changes. Recent developments of generalized PPI may, however, be useful to probe FC changes in the whole brain (McLaren et al., 2012). Thirdly, it cannot be excluded that ongoing intrinsic connectivity during a task may have produced the match between resting-state and task-related networks. As mentioned previously task-related network activity may be a mixture of task-related network activation and basic “resting-state” activity of a network (Fox et al., 2007). The current approach could not disentangle these two sources entirely. Furthermore, local activity is probably the results of multiple networks rather than a single network (Xu et al., 2013). The current approach aimed at pair-wise matches between resting-state and task-related components, which may pose a simplification of the additive effects of multiple networks. Future studies may address these complexities. Fourth, some participants showed SCD, which may show increased likelihood to progress to Alzheimer’s dementia (Jessen et al., 2014).

## Conclusion

We could show that specific networks are specifically activated during successful episodic memory and are also present during resting-state. The level of connectivity within these networks during resting-state was, however, not predictive of the level of task-related activation.

## Author Contributions

LS-V collected the MRI data, performed the analysis, and wrote the manuscript draft. AT, MAC, and NF collected the MRI data and performed the analysis. KB, CC, DJ, BE-W, and MD collected the data and critically revised the manuscript. LK-I collected the data, developed the fMRI paradigm, and critically revised the manuscript. ME collected the data, developed the hypotheses, wrote the manuscript, and supervised the data analysis.

## Conflict of Interest Statement

The authors declare that the research was conducted in the absence of any commercial or financial relationships that could be construed as a potential conflict of interest.

## Footnotes

1 http://www.facevar.com/glasgow-unfamiliar-face-database

2 http://corpora.informatik.uni-leipzig.de

3 http://mialab.mrn.org/software/gift/

4 https://www.R-project.org/
