# Supplementary material for: Correspondence Between Resting-State and Episodic Memory-Task Related Networks in Elderly Subjects
Source: Front Aging Neurosci. 2018 Nov 8;10:362. doi: 10.3389/fnagi.2018.00362 (PMC6236026; doi:10.3389/fnagi.2018.00362)
Supplement: Supplementary file 1 [file Data_Sheet_1.docx]

Supplementary Figure 1:

The results are displayed on representative sections of the 152 MNI template (1mm resolution). The task-related networks are thresholed at z>2 and binarized and the rsfMRI were thresholded at z>3 and binarized. The best matching rsfMRI component (IC 9) that best matched the task-related component is shown in the top row. The second best matching rsfMRI component (IC 3) is shown in the second row. For the second best matching component, note the mismatch in regions such as the lateral occipital cortex, hippocampus and parahippcampus.
